# Effect of *Silene vulgaris* and Heavy Metal Pollution on Soil Microbial Diversity in Long-Term Contaminated Soil

**DOI:** 10.1007/s11270-017-3655-3

**Published:** 2017-12-21

**Authors:** Magdalena Pacwa-Płociniczak, Tomasz Płociniczak, Dan Yu, Jukka M. Kurola, Aki Sinkkonen, Zofia Piotrowska-Seget, Martin Romantschuk

**Affiliations:** 1 Department of Microbiology, University of Silesia, Jagiellońska 28, 40-032 Katowice, Poland; 2 Department of Environmental Sciences, University of Helsinki, Niemenkatu 73, 15140 Lahti, Finland; 3 Institute of Environmental Sciences, Kazan Federal University, Kremlevskaya 18, 420008 Kazan, Russia

**Keywords:** Heavy metals, Soil pollution, *Silene vulgaris*, Bacterial diversity, Real-time PCR

## Abstract

In this study, we analysed the impact of heavy metals and plant rhizodeposition on the structure of indigenous microbial communities in rhizosphere and bulk soil that had been exposed to heavy metals for more than 150 years. Samples of the rhizosphere of *Silene vulgaris* and non-rhizosphere soils 250 and 450 m from the source of emission that had different metal concentrations were collected for analyses. The results showed that soils were collected 250 m from the smelter had a higher number of Cd-resistant CFU compared with the samples that were collected from 450 m, but no significant differences were observed in the number of total and oligotrophic CFU or the equivalent cell numbers between rhizosphere and non-rhizosphere soils that were taken 250 and 450 m from the emitter. Unweighted pair group method with arithmetic mean (UPGMA) cluster analysis of the denaturing gradient gel electrophoresis (DGGE) profiles, as well as a cluster analysis that was generated on the phospholipid fatty acid (PLFA) profiles, showed that the bacterial community structure of rhizosphere soils depended more on the plant than on the distance and metal concentrations. The sequencing of the 16S rDNA fragments that were excised from the DGGE gel revealed representatives of the phyla *Bacteroidetes*, *Acidobacteria*, *Gemmatimonadetes*, *Actinobacteria* and *Betaproteobacteria* in the analysed soil with a predominance of the first three groups. The obtained results demonstrated that the presence of *S. vulgaris* did not affect the number of CFUs, except for those of Cd-resistant bacteria. However, the presence of *S. vulgaris* altered the soil bacterial community structure, regardless of the sampling site, which supported the thesis that plants have a higher impact on soil microbial community than metal contamination.

## Introduction

Industrial development and mining activities have led to a significant environmental heavy metal contamination in many locations around Europe. One such polluted area is Upper Silesia in Southern Poland where several ferrous and non-ferrous metal processing plants have emitted large amounts of heavy metals such as Cd, Zn, Cu and Pb for more than 150 years. For example, the concentrations of Cd, Pb and Zn in soils in this region can reach 90.8, 394.7 and 8403.3 mg kg^−1^, respectively (Nadgórska-Socha et al. 2015). Heavy metals have been found to have serious consequences for the soil ecosystem at these or even lower concentrations. Only a relatively small number of plant species, called metallophytes, are capable of surviving and reproducing in such environments. In the study area, *Festuca ovina*, *Silene vulgaris* and *Arabidopsis arenosa* were the only plant species found.

Soil contaminated with heavy metals not only provides sources of specific plants but also of metal-tolerant microorganisms. It is known that, in general, heavy metals negatively affect the number and biomass of soil microorganisms (Kelly et al. 2003; Renella et al. 2012; Oliveira and Pampulha 2006) as well as their activity (Kandeler et al. 1996; Wang et al. 2007) and diversity (Sandaa et al. 1999; Li et al. 2006; Ruyters et al. 2012).

However, some metal-tolerant bacteria, especially those that are known as plant growth-promoting bacteria, are of great importance for the stimulation of plant development. There is also evidence that such bacteria support the phytoremediation of soil that has been contaminated with heavy metals (Belimov et al. 2005; Płociniczak et al. 2013; Jing et al. 2014, Wu et al. 2016a; Ye et al. 2017). The presence of metal-tolerant bacteria in contaminated soil results from their capacity to grow in environments with toxic concentrations of heavy metals. Microbes can adapt to heavy metals by means of a cell’s intrinsic properties such as changes in the cell wall structure, extracellular polysaccharide production and the ability to bind or precipitate metals inside or outside the cell. Moreover, some microorganisms may possess genes that encode highly specific mechanisms of resistance to heavy metals (Nies and Silver 1995; Bruins et al. 2000). These genetic determinants can be chromosomal or located on plasmids (Cervantes and Gutierrez-Corona 1994; Wuertz and Mergeay 1997). The localisation of these systems on plasmids and other mobile genetic elements allows the spread of the genes that are responsible for metal resistance among soil microbial populations. In fact, the results of several studies have shown that elevated metal concentrations can change the microbial community structure accompanied by an increase in the abundance of metal-tolerant strains (Hui et al. 2012).

The aim of this study was to examine the selective pressure of heavy metals and the impact of rhizodeposition on the structure of the microbial communities on sites that varied in heavy metal concentrations that were selected based on our previous investigations. Since root exudates affect the bioavailability of heavy metals for bacterial strains and plants, we analysed bulk soil and the rhizospheres of *S. vulgaris*, the dominant plant species at the sites at the time of sampling that had been exposed to emission from a non-ferrous smelter for many years. In order to achieve the goal of the study, the communities of two bulk soils and rhizospheres (taken within distances of 250 and 450 m from the source of the emission) were assessed using phospholipid fatty acid (PLFA) analysis, polymerase chain reaction-denaturing gradient gel electrophoresis (PCR-DGGE) and DNA sequencing of significant rybotypes from DGGE gel. In addition, the plate dilution method and real-time PCR were used to enumerate the culturable and total bacterial fractions, respectively. The culturable Cd-resistant bacteria were isolated and identified using 16S rDNA sequencing.

We hypothesised that (1) metal-resistant/metal-tolerant bacterial strains are common in long-term contaminated soil, (2) plants have a higher impact on a soil microbial community than metal contamination and (3) that microbial communities in plant-free plots do not form a homogeneous group when compared with plots where *S. vulgaris* is present.

## Materials and Methods

### Study Area and Sampling

The study site is located in Katowice, Upper Silesia (Southern Poland), close to the non-ferrous smelter “Szopienice”. As a result of the emissions from this non-ferrous smelter, heavy metals have accumulated in this area for more than 150 years. Two composite soil samples from a depth of 10–20 cm (each prepared from eight different subsamples taken from an area of 25 m^2^) were collected at distances of 250 m (NR250) and 450 m (NR450) from the source of the emissions. In addition to the bulk soil, rhizosphere samples of the bladder campion (*S. vulgaris*) were collected. At three random spots (about 250 and 450 m from the emitter), four *Silene* plants (with the distance between them of about 4 m) from each sampling site (10 × 10 m) and their associated root material were harvested by removing a 1-l soil core. The roots were recovered from the soil cores, gently shaken to remove any loosely adhering soil, placed in a sterile plastic bag and transported at 4 °C. In the laboratory, roots from the four plants were separated from the shoots using a sterile scalpel and the root-adhered soil from the four plants was pooled together, thus giving a mixed sample of rhizosphere soil for each collection point (R250 and R450). The soils used in the study were classified as sandy soils. Their detailed chemical and physical parameters are listed in Table 1.

**Table 1:** Selected physicochemical properties of soils used in the experiment

Properties	NR250	NR450
Sand (2–50 μm) (%)	92^a^	98^a^
Silt (50–2 μm) (%)	8^a^	8^a^
Clay (< 2 μm) (%)	0^a^	3^a^
Textural classification	Sandy	Sandy
Density (g cm^−3^)	1.64^a^	1.57^a^
pH_H2O_	7.75^a^	7.65^a^
pH_KCl_	6.84^a^	7.01^a^
Soil moisture content (%)	2.12^a^	1.95^a^
Organic matter (g kg^−1^)	22.15^a^	19.05^a^
N (%)	0.09^a^	0.08^a^
C (%)	1.39^a^	1.42^a^

Values marked with different superscripted letters differ significantly at *P* < 0.05 in an ANOVA followed by Bonferroni post hoc tests (*P* < 0.05)

### Chemical Analyses

The soil samples were analysed for total and water-extractable heavy metal concentrations. In order to determine the total metal concentrations, triplicate subsamples (1 g) of each soil dried to a constant weight at 105 °C were digested in a mixture of 65% HNO_3_ (4 ml), 30% H_2_O_2_ (2 ml) and distilled H_2_O (1 ml) using an MLS 1200 MEGA microwave oven (Milestone, USA). The mineralisation was performed according to the method recommended by the manufacturer. The total concentrations of Zn, Cu, Cd and Pb were estimated using an atomic absorption spectrometer (UNICAM 939/959). To measure the water-extractable content of the metals, triplicate soil samples (5 g) were suspended in 50 ml of double distilled H_2_O and shaken for 2 h at 180 rpm. After filtration (0.22 μm), the concentrations of the metals were determined using the same device.

### Quantification of Culturable Soil Bacteria

In order to extract bacterial cells from the bulk soil and the rhizosphere, triplicate composite soil samples (5 g) or roots with adhering rhizosphere soil were placed in Erlenmeyer flasks containing 45 ml of 0.85% NaCl and shaken at 180 rpm for 30 min. Then, serial ten-fold dilutions of the soil suspensions were plated onto agar plates: broth agar, 0.1-strength trypticase soy agar (TSA) and 0.1-strength TSA supplemented with 1 mM of Cd to enumerate the total culturable bacteria and the oligotrophic and Cd-tolerant fraction of those bacteria, respectively. In order to inhibit the growth of fungi, cycloheximide at a concentration of 100 μg ml^−1^ was added to the medium. The plates were incubated at 24 °C for 5 days prior to counting the CFU.

### Isolation and Identification of Cd-Resistant Bacteria

Cd-resistant bacterial colonies that were grown on 0.1-strength TSA supplemented with 1 mM of Cd were purified on a medium containing 1 mM of Cd and then identified by sequencing sections of the 16S rRNA gene. For this purpose, an individual bacterial colony was suspended in 25 μl of sterile water, boiled (10 min), centrifuged for 10 min at 13,000 rpm and the supernatant was used as the template for PCR using the universal bacterial primers MF341 (5′- CCT ACG GGA GGC AGC AG-3′) (Muyzer et al. 1993, modified) and MR907 (5′-CCG TCA ATT CMT TTG AGT TT-3′) (Ishii et al. 2000, modified) in order to target the V3–V5 region of the 16S rRNA gene, which is about 570 bp in size.

### DNA Extraction

Each tested soil was split into three subsamples and then DNA was extracted directly from 0.3 g (fresh weight) of each soil subsample using a Fast DNA® SPIN kit for soil (Qbiogene Inc., Carlsbad, USA) according to the manufacturer’s instructions. Each DNA subsample was treated as a replicate. The concentration of DNA was measured fluorometrically using a PicoGreen® dsDNA Quantitation Kit (Molecular Probes Inc., Eugene, OR, USA) as specified by the manufacturer’s instructions. In order to ensure representativeness, the samples consisted of multiple subsamples of well-mixed soil.

### Culture-Independent Quantification of the Bacterial Communities by Quantitative PCR

For the quantification of the equivalent cell numbers, real-time PCR of a fragment of 16S rRNA gene was performed using specific primers pE (5′-AAA CTC AAA GGA ATT GAC GG-3′) and pF^•^ (5′-ACG AGC TGA CGA CAG CCA TG-3′) (Edwards et al. 1989) in 20-μl reaction mixtures using buffers supplemented with SYBRGreen PCR Mix (Finnzymes Oy, Espoo, Finland), 0.2 μM of each primer and 2 μl of 50-fold diluted DNA. The procedure for the real-time PCR followed the method described by Hermansson and Lindgren (2001) and Wu et al. (2016b) with minor modifications. The template DNA was amplified and monitored using an Opticon III instrument (MJ Research Inc., Waltham, MA, USA). The amplification was run with a temperature programme of 10 min at 94 °C and 30 cycles of 10 s at 94 °C followed by annealing for 20 s at 57 °C and extension for 30 s at 72 °C. Fluorescence data were acquired at the end of each extension step at 81 °C in order to avoid the detection of primer dimers. For the melting curve analysis of the products, the temperature was raised from 65 to 95 °C and the melting temperatures were determined using Opticon III software. The amplification efficiency was determined by the serial dilution of DNA with known equivalent cell numbers that was extracted from *Nitrosomonas europea* (ATCC 19718), followed by real-time PCR. The quantification of 16S rRNA gene copies was based on the mean slope value derived from the standard curve. Enumeration of equivalent cell numbers was performed by dividing the 16S rRNA gene copies that were obtained by 4.2 (the average of 16S rRNA gene copies per bacterial genome) (Case et al. 2007).

### PCR-DGGE

The universal bacterial primers MF341-GC (5′-CGC CCG CCG CGC CCC GCG CCC GTC CCG CCG CCC CCG CCCG CCT ACG GGA GGC AGC AG-3′—GC clamp underlined) (Muyzer et al. 1993, modified, Muyzer and Smalla 1998) and MR907 (5′-CCG TCA ATT CMT TTG AGT TT-3′) (Ishii et al. 2000), which target the V3–V5 region of the 16S rRNA gene were used to amplify fragments of about 570 bp. The PCR were run with a mixture containing 1 μl of the ten-fold diluted DNA template, 0.2 μM of each primer, a 10× reaction buffer (DyNAzyme, including 1.5 mM MgCl_2_, Finnzymes), 200 μM of dNTP and 1 U of DyNAzyme II DNA polymerase (Finnzymes) in a PTC-100 Thermo Cycler (MJ Research Inc., Walthman, MA, USA). PCR amplification was performed at 94 °C for 5 min and 37 cycles of 20 s at 94 °C followed by annealing for 20 s at 56 °C and an extension step of 30 s at 72 °C. DGGE analysis of the amplified products was performed using a DCode™ Universal Mutation Detection System (Bio-Rad, Hercules, CA, USA). The PCR products (30 μl, containing 100 ng of DNA) were loaded onto 9% (*w*/*v*) polyacrylamide gels in a 1× Tris-acetate-EDTA buffer (pH 7.4) containing a linear denaturing gradient from 35 to 70% (100% denaturant solution contained 7 M urea and 40% deionised formamide). Gels were run at 200 V, 60 °C for 30 min and then at 80 V, 60 °C for 17 h. After electrophoresis, gels were stained with a SYBR®Gold nucleic acid gel stain (10,000-fold diluted with a 1× TAE buffer, Molecular Probes) for 30 min and photographed using a gel photo system (ChemiImager 5500 v. 3.1, Alpha Innotech Co., San Leonardo, CA, USA).

DGGE profiles were transformed into binary code with each band position scored as 1 (present) or 0 (absent). The pairwise similarity of the banding patterns of the different samples was calculated by applying the Nei-Li distance (Nei and Li 1979) and an unweighted pair group method with arithmetic mean (UPGMA) cluster analysis was carried out using the DGGEstat program (van Hannen, the Netherlands Institute for Ecological Research, NIOO-KNAW, the Netherlands). Bootstrap values (100 replications) were calculated in dendrograms to evaluate the reliability of each group (cluster).

### DNA Sequencing and Phylogenetic Analyses

For sequence analysis, single bands were cut out from the DGGE gel, crushed in Eppendorf tubes containing 20 μl of distilled water and kept overnight at 4 °C. This step was repeated several times. Three microlitres of the eluted DNA was used as a template for PCR amplification with the universal primers MF341 (without the GC clamp) and MR907 as described. The sequencing reactions were performed using an ABI PRISM® BigDye™ Terminator Cycle Sequencing Ready Reaction kit and analysed on an ABI Prism 3700 DNA sequencer (Applied Biosystems, Foster City, CA, USA). This analysis was also used for the identification of Cd-resistant isolates. All sequence chromatograms were analysed using the Staden Package (University of Cambridge, UK). The sequences were compared with known 16S rRNA gene sequences in the EMBL database using a BLAST server at the European Bio-Informatics Institute (EBI; http://www.ebi.ac.uk, Hinxton Hall, Cambridge, UK). DNA sequences were aligned using CLUSTAL W (Thompson et al. 1994). Phylogenetic analyses were performed using the neighbour-joining (NJ) method to test the support for the phylogeny with a bootstrap analysis based on 1000 replicates using MEGA ver. 6.0 (Kumar et al. 2008).

### Nucleotide Sequence Accession Numbers

The partial 16S rRNA gene sequences that were determined in this study have been deposited in the GenBank nucleotide sequence database under accession numbers HE588188 to HE588205.

### Phospholipid Fatty Acid Analysis

PLFAs were extracted as described by (Pennanen et al. 1999) with minor modifications. Briefly, 2-g fresh weight of a soil sample was extracted with a chloroform:methanol:citric buffer (0.15 M) mixture (1:2:0.8 *v*/*v*/*v*) and the lipids were fractionated into neutral lipids, glycolipids and phospholipids in silicic acid columns (Supelco). The phospholipids were subjected to mild alkaline methanolysis and the fatty acid methyl esters were separated with a gas chromatograph (Hewlett-Packard 6890, USA) using a capillary column HP-Ultra 2 (cross-linked 5% phenyl-methyl silicone; 25 m, 0.20 mm ID; film thickness 0.33 μm) with hydrogen as the carrier gas. Fatty acid methyl ester (FAME) compounds were detected using a flame ionisation detector (FID) and identified using the MIDI Microbial Identification System software (Sherlock TSBA40 method and the TSBA40 library; MIDI Inc., Newark, DE, USA).

### Statistical Analyses

Statistical analyses of the total and water-extractable concentrations of heavy metals and microbial counts (log transformed) were performed for each soil and were subjected to an analysis of variance (ANOVA) and post hoc comparison of the means using the Bonferroni (equal variances) or Tamhane’s T2 (unequal variances) tests (GLM Univariate in SPSS 19.0, SPSS Inc., Cary, NC, USA). In addition, *t* tests were used when appropriate. Cluster analysis of the PLFA profiles was performed on Euclidean distances using the PAST data analysis package with the default settings (Hammer et al. 2001). The species diversity values (*H*) that were calculated from the PLFA and DGGE data from five soils were compared by Pearson correlation (Statistica 9.0 PL). The assumptions of the analyses were met.

## Results

The total concentrations of Pb, Cd, Cu and Zn in the tested soils were high and depended on the distance from the smelter (Table 2). However, the water-extractable fractions of Cd, Cu, Zn and Pb constituted only a small portion of the total metals. The water-extractable Cd constituted from 5.8% (NR250) to 10.6% (R450) of the total Cd concentration in the soil, while the water-extractable fraction of Pb was much lower and only reached values of 0.8 to 2.4%. Compared with Cu, Zn and Pb, Cd was characterised by a greater water extractability, which indicates that a higher portion of the total concentration was also bioavailable.

**Table 2:** Total and water-extractable concentrations of heavy metals (mg kg^−1^)

Sample	Cd		Cu		Zn		Pb	
	Total	W-E	Total	W-E	Total	W-E	Total	W-E
R250	86.24	7.47	131.23	6.12	8109.85	248.62	695.96	5.73
NR250	111.11	6.41	158.95	4.24	10,147.60	234.24	1104.50	9.50
R450	45.12	4.78	74.25	4.21	5487.32	196.48	527.40	6.39
NR450	42.14	3.54	90.11	2.84	6003.50	164.55	917.90	7.36

*W-E* water-extractable

Increasing the distance from 250 to 450 m from the smelter decreased (*p* < 0.0005) and the presence of rhizosphere increased (*p* = 0.096) the number of Cd-resistant CFU (*F* = 19.2, df = 12, 3, *p* = 0.001). The number of bacteria that were resistant to Cd was a magnitude higher in samples R250 and NR250 as compared with soils R450 and NR450. In soils R250 and NR250, the Cd-resistant fraction constituted 88.3 and 95.5% of the oligotrophic colony-forming bacteria, respectively. In contrast, in both the R and NR soils that was taken 450 m from the source of the emission, the Cd-resistant bacteria constituted about 8.5% of the total oligotrophic colony formers (Table 3). Neither the presence of a rhizosphere nor distance from the smelter affected the number of total CFU, oligotrophic CFU or the number of equivalent cell number (*p* > 0.2). As was expected, the equivalent cell number calculated by real-time PCR was higher compared with that of the bacterial counts that were obtained by plating (Table 3). The culturable bacterial fraction that was calculated in the soils constituted between 0.6 and 3.1% of the equivalent total cell number.

**Table 3:** Numbers of total culturable, equivalent cell number, oligotrophic and Cd-resistant bacteria

Sample	Total CFU g^−1^ of soil	Equivalent cell number g^−1^ of soil^a^	Oligotrophic CFU g^−1^ of soil	Cd-resistant CFU g^−1^ of soil
R250	5.3 × 10^6^a	2.0 × 10^8^a	2.6 × 10^5^a	2.4 × 10^5^a
NR250	1.1 × 10^6^a	1.7 × 10^8^a	1.3 × 10^5^a	1.3 × 10^5^b
R450	7.2 × 10^6^a	4.7 × 10^8^a	4.4 × 10^6^a	3.8 × 10^4^c
NR450	4.7 × 10^6^a	1.5 × 10^8^a	3.3 × 10^5^a	2.9 × 10^4^c

Values marked with different letters differ significantly at *P* < 0.05 in an ANOVA followed by Bonferroni post hoc tests

^a^16S rRNA gene copies divided by the average of 16S rRNA gene copies per bacterial genome

**Fig. 1:**
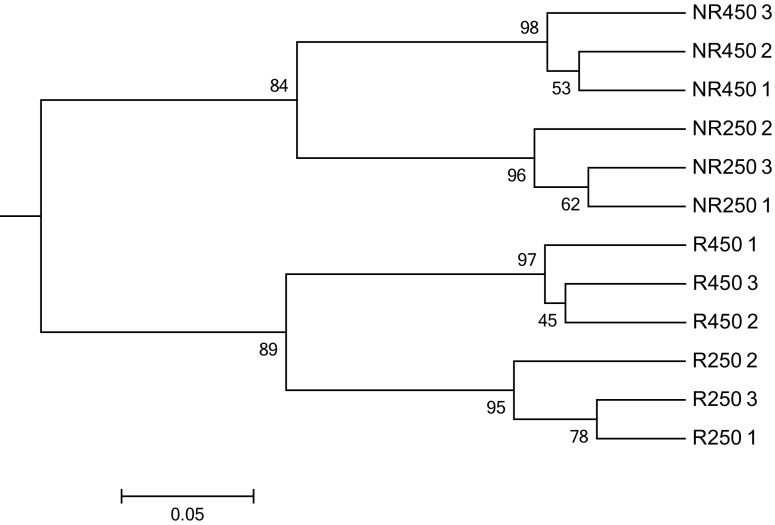
UPGMA cluster analysis based on the similarity between DGGE profiles for of PCR-amplified 16S rRNA gene fragments of bacterial communities from analysed soils. Bootstrap values from 1000 replications are indicated at the branches

PCR-DGGE analysis was used to characterise the dominant bacteria in the soil. The highest number of visible bands was detected for the R450 and NR450 soils (24), and then for the R250 (22) and NR250 soils (20). In all of the profiles that were derived from the tested soils, bands containing DNA fragments with a high GC content (i.e. visible in the lower part of the gel) were detected in all of the profiles that were analysed. The dendrograms that were created from the DGGE profiles of PCR-amplified 16S rRNA gene fragments show a division of the samples into two groups based on the structure of their bacterial communities. The first group included the samples from rhizospheric soils (R250 and R450) and the second group consisted of the non-rhizospheric samples NR250 and NR450 (Fig. 1).

**Fig. 2:**
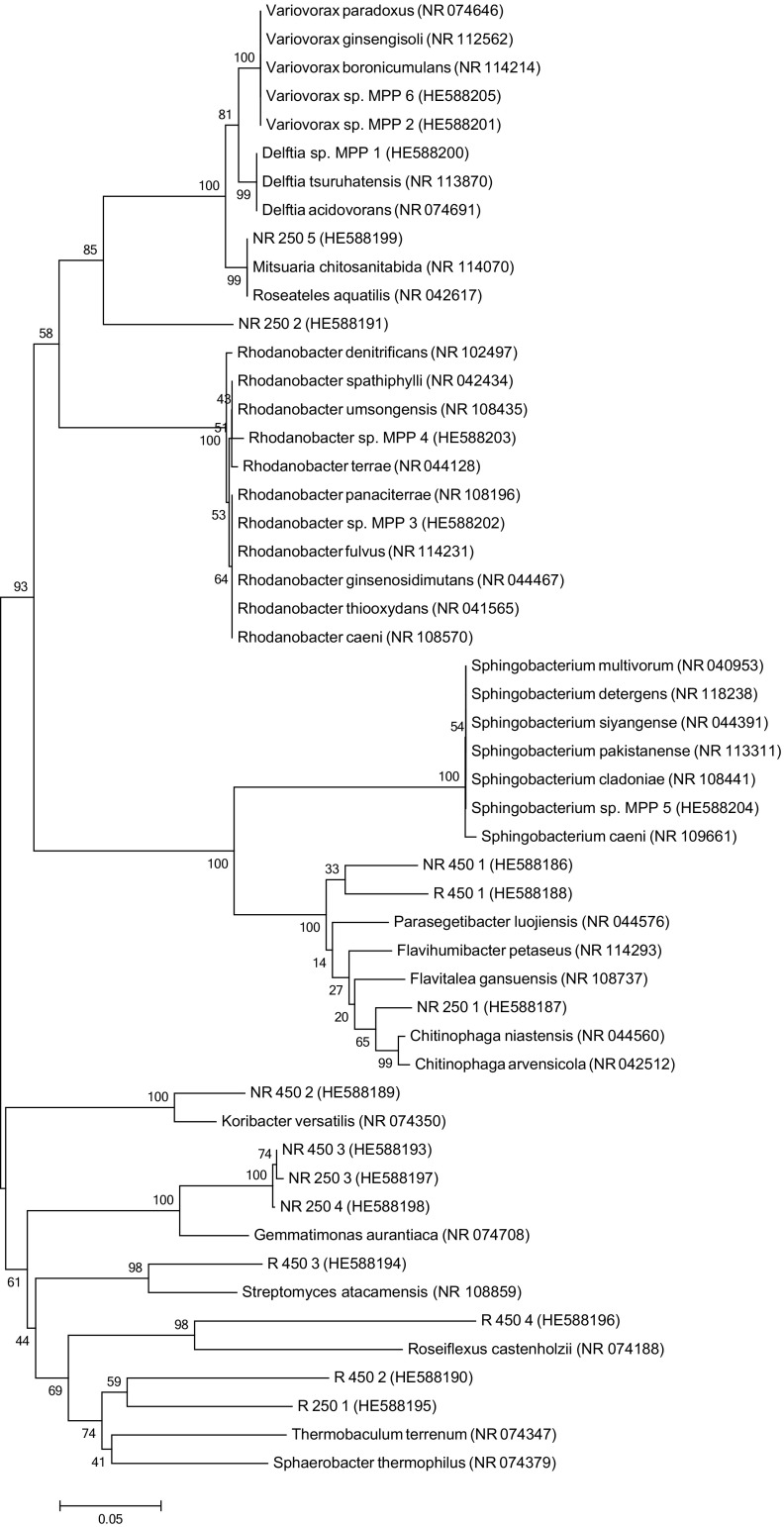
Neighbour-joining phylogenetic tree of bacteria based on 16S rRNA gene sequence comparisons. Bootstrap values from 1000 replications are indicated at the branches. GenBank accession numbers are given in parentheses; *Delftia* sp. MPP 1 (HE588200), *Variovorax* sp. MPP 2 (HE588201), *Rhodanobacter* sp. MPP 3 (HE588202), *Rhodanobacter* sp. MPP 4 (HE588203), *Sphingobacterium* sp. MPP 5 (HE588204), *Variovorax* sp. MPP 6 (HE588205) - cultured bacteria

Representatives of the DNA bands that were unique for each soil were excised from the PCR-DGGE banding patterns, reamplified and subjected to DNA sequencing. A total of 13 DNA fragments excised from DGGE gel were sequenced. Moreover, DNA sequencing was carried out in order to identify the six dominant Cd-resistant isolates that were cultured on a 0.1-strength TSA medium supplemented with 1 mM of Cd. The phylogenetic relationships of the PCR-DGGE retrieved 16S rDNA sequences and the Cd-resistant isolates are shown in Fig. 2. The 13 sequences retrieved from the DGGE belonged to the phyla *Bacteroidetes*, *Acidobacteria*, *Actinobacteria*, *Chloroflexi*, *Gemmatimonadetes* and *Betaproteobacteria*. The DNA fragments that migrated to similar positions in the DGGE gel were identified as the same phyla. The six dominant Cd-resistant isolates were represented by *Beta*- and *Gamma-proteobacteria* and *Bacteroidetes* and were related to *Delftia* sp., *Variovorax* sp., *Rhodanobacter* sp. and *Sphingobacterium* sp., respectively.

**Fig. 3:**
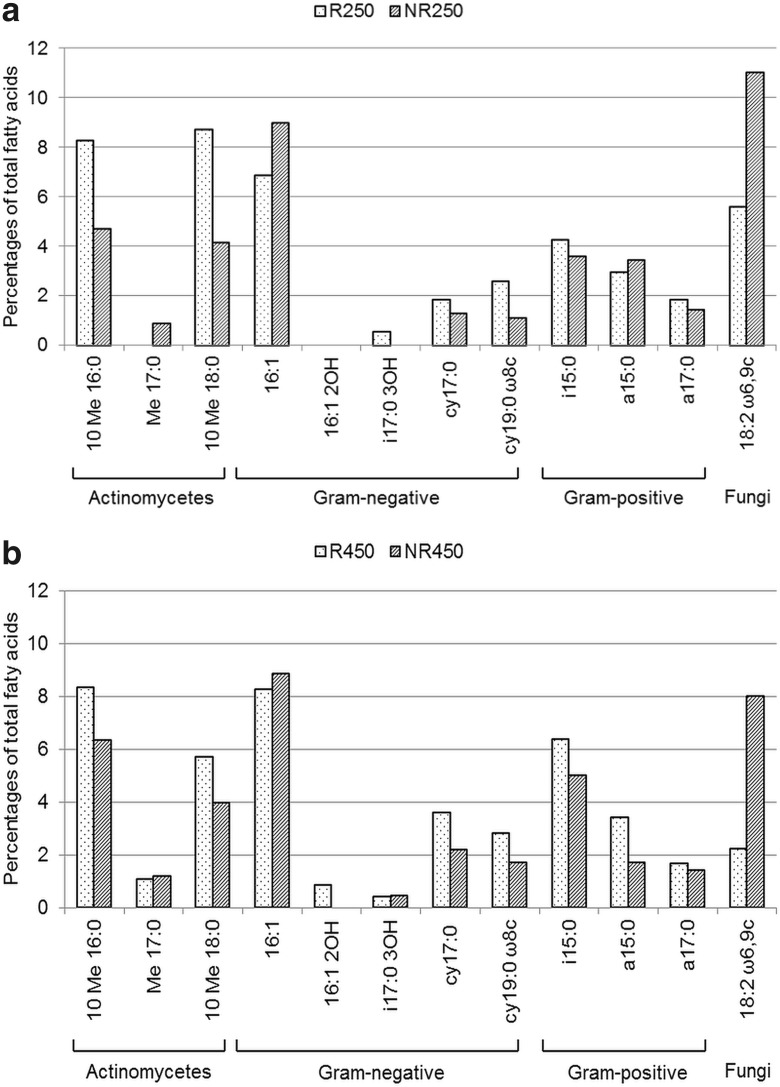
Abundance (% of total fatty acids) of chosen PLFAs in the R and NR soils sampled 250 m (**a**) and 450 m (**b**) from the source of emission

PLFA analysis was used to characterise the dominant microbial groups in the soil. The results showed that the PLFA profiles of the tested rhizosphere and non-rhizosphere soils with different metal concentrations contained several similar types of fatty acids (Fig. 3). Methylated 17:0 fatty acids and two of the hydroxyl fatty acids (16:1 2OH, 17:0 3OH) were the exceptions. The Me 17:0 was found in soils NR250, R450 and NR450, but was not observed in R250 sample. Hydroxylated 16:1 2OH was detected only in R450, while 17:0 3OH was present in all of the samples (R and NR) that were taken 450 m from the emitter and in the rhizosphere soil at 250 m. The fatty acids that are characteristic for Gram-positive bacteria were detected in all of the samples. The following fatty acids 10 Me 16:0, 10 Me 18:0, cy17:0, cy19:0ω8c, i15:0 and a17:0 were isolated from all of the soils tested, but at higher concentrations in the rhizosphere soil. The abundance of the fungal biomarkers 18:2ω6,9c was higher in the NR than that in the R samples and higher in the samples that were collected 250 m from the source of contamination than in those that were collected farther away.

The summed percentages of group-specific fatty acids for actinomycetes, Gram-positive, Gram-negative and fungi are presented in Table 4. The proportions of fatty acids that are characteristic for *Actinomycetales* were higher in the rhizosphere soils (R250 and R450) than in the non-rhizosphere soils (NR250 and NR450) (*t* = 4.9, *p* = 0.039). In the case of the indicator fatty acids that are characteristic for Gram-negative bacteria, no difference was observed between the soils that were sampled 250 m from the emitter (*t* = 1.1, *p* = 0.38). However, Gram-negative bacteria had a lower abundance in the 250-m samples than in the samples that were taken 450 m from the emitter (*t* = −3.5, *p* = 0.013). The percentage distribution of fungal indicator fatty acids was higher in the non-rhizosphere soils (NR250 and NR450) than that in the rhizosphere of *S. vulgaris* (*t* = 3.6, *p* = 0.011). Cluster analysis that was generated on the PLFA profiles showed a separation of the microbial communities into two groups in the same way as the DGGE dendrogram (Fig. 1). Two groups were distinguished—the first included the R250 and R450 soils and the second the NR soils from 250 and 450 m. The species diversity values (*H*) that were calculated from the DGGE and PLFA data significantly correlated (*r* = 0.75, *n* = 4, *p* < 0.01) between the soil-specific mean values obtained by these two methods.

**Table 4:** Summed percentages of chosen microbial PLFA biomarkers characteristic for the main group of microorganisms in tested soils

Soil	*Actinomycetes*	GN	GP	Fungi
R250	17.01	11.92	9.12	5.63
NR250	9.77	11.43	8.54	11.04
R450	15.19	16.01	11.50	2.24
NR450	11.53	13.24	8.17	8.01

*GN* Gram-negative, *GP* Gram-positive

## Discussion

Contamination of soil with heavy metals has an indisputable impact on the structure and biodiversity of the microbial communities inhabiting polluted environments (Yao et al. 2003; Vishnivetskaya et al. 2011; Deng et al. 2015; Xie et al. 2016). However, the impact of different concentrations of these pollutants on the microbial communities inhabiting the rhizosphere of metallophytes remains unclear and is, therefore, the subject of the presented research. It is well-known that the structural and functional diversity of the rhizosphere is dependent on the root exudates that stimulate the growth of specific bacterial and fungal populations (Smalla et al. 2001). It was, therefore, interesting to examine whether the microbial diversity within the rhizosphere was subject to the changes that are induced by the presence of heavy metals, as it was observed in the case of bulk soils, or whether the structure of microbial communities is determined by the presence of a plant.

In this study, the rhizosphere and non-rhizosphere soil samples that were collected at distances of 250 and 450 m from the emitter contained total concentrations of Cd, Zn and Pb that exceed the maximum accepted values in Europe (Council of the European Communities 1986). The water-extractable fractions of these heavy metals represented only a few percent of their total concentrations, thus indicating that the soil strongly limited the availability of metals.

The samples that were taken 250 m from the emitter had a higher number of Cd-resistant bacteria and the concentration of Cd was also higher compared with that of the samples that were collected 450 m from the source of contamination. Rather, we did not observe significant differences between the number of total and oligotrophic CFU or the equivalent cells in the rhizosphere and non-rhizosphere soils that were collected 250 and 450 m away from the emitter. These results reflect the selection of metal-resistant bacteria in soils that are under long-term exposure to heavy metals. As these results concerned the culturable fraction only, which consists of about 1% of the total bacteria that inhabit soils, and as the limitations of the cultivation approach for enumeration of bacteria are well-known (Tebbe et al. 1992; Rathnayake et al. 2013), it is not guaranteed that these results can be generalised to non-cultivable microbes. Despite these limitations, the CFU results support the hypothesis that heavy metal contamination has affected the microbial community of both rhizosphere and bulk soil in the vicinity of the non-ferrous metal works. In accordance with our results with Cd, Ryan et al. (2005) found that Zn-, Cu- and As-resistant bacteria dominated a Zn-, Cu- and As-contaminated site. Similar to our results, Sułowicz et al. (2011) observed a high percentage of metal-tolerant bacteria in both the non-rhizosphere and rhizosphere soil of silver birch and bush grass that were taken from a metal-mine heap in Piekary Slaskie (Upper Silesia, Poland). Our findings are also in agreement with the results reported by Müller et al. (2001), who observed that in soil localised 1 m from the centre of Hg contamination, the number of metal-resistant bacteria was 25 times higher than that in soil that was collected 19 m from the emitter and contained 20 times less Hg in soil. However, Renella et al. (2012) did not observe any changes in the number of bacteria from different physiological groups nor in the number of Cd-resistant bacteria in both soils that had been planted with maize and non-planted Cd-contaminated soils. Furthermore, they concluded that high Cd concentrations primarily induced physiological adaptations rather than a selection for metal-resistant bacteria in contaminated soil.

Interestingly, even though the UPGMA cluster analysis of the DGGE profiles of soil samples as well as the cluster analysis that was generated on the PLFA profiles indicated that the bacterial community structure of rhizosphere soils depended more on the plant than on the distance, i.e. the total level of metal contamination, the abundance of bacteria (as CFUs) was not higher in the rhizosphere of *S. vulgaris*, as compared with that of soil samples taken from bare soil at the same contaminated sites. *S. vulgaris*, the dominant plant at the sites that was analysed, is a pseudometallophyte that is able to accumulate heavy metals in its roots and plant tissues (Mohtadi et al. 2012), which may affect the bacterial community structure in the vicinity of roots that are rich in toxic metals. In addition, plants can alter the chemical mobility of heavy metals by producing root exudates, especially organic acids (Wang et al. 2004; Feng et al. 2005; Rajkumar et al. 2012) and thus a higher concentration of water-extractable metals in the rhizosphere of *S. vulgaris* had impact on the microbial community structure. Moreover, the roots of plants exude various carbon compounds that select for microorganisms that are specialised in their degradation. On the other hand, some of the plant antimicrobial compounds, e.g. phytoalexins, change the composition of a rhizosphere microbial community (Wood et al. 2016; Płociniczak et al. 2017). In contrast to our observation, Deng et al. (2015), who studied the bacterial community dynamics in response to different metal concentrations in the rhizospheres of rape and paddy, found that the DGGE profiles of 16S rRNA gene fragments grouped according to the sampling site and not in the relation to the plant of which they were collected.

The influence of *S. vulgaris* on the microbial community diversity in the studied soils was also detected from PLFA analysis. Rhizosphere-induced changes to the microbial community structure were accompanied by a decrease in the summed percentages of fungal PLFAs and an increase in the percentages of the fatty acids that are characteristic for *Actinomycetes* and Gram-positive bacteria. This indicates that the abundance of specific microbial groups was different between the rhizosphere and non-rhizosphere samples, even though the total amount of CFUs was not statistically different. The reason for the lack of the latter difference might be the fact that the CFUs are cultivated on plates rather than being a direct measure of bacterial abundance in soil.

Contamination of soil by heavy metals alters the structure of soil microbial communities, e.g. by changing the relative abundance of some microbial PLFAs (Pennanen et al. 1996; Xie et al. 2011; Azarbad et al. 2013). In our study, all of the soils were dominated by fatty acids, which are thought to be markers of Gram-negative bacteria. Earlier, a similar dominance of Gram-negative bacteria was observed in soils that had been artificially contaminated with different heavy metals (Frostegård et al. 1993) in the soil around a Zn smelter (Kelly et al. 2003) and in the soil of the metal-mine spoil heap (Sułowicz et al. 2011).

In contrast, Kozdrój and van Elsas (2001) found a high percentage of branched fatty acids using the FAME approach for their study of structural diversity in a heavily industrialised area, thus indicating the dominance of Gram-positive bacteria in the microbial communities that were studied. The presence of *Actinomycetales*, as revealed by methyl-branched phospholipid fatty acids (10Me16:0, Me17:0, 10Me18:0), has been reported to be significant in metal-impacted soils (Gremion et al. 2003) although the distribution of these bacteria depends on the type of soil (Thompson et al. 1994). Our investigation showed a high total concentration of methyl-branched PLFAs (10Me16:0, 10Me17:0, 10Me18:0) and the presence of three (in the NR250, R450 and NR450 soils) or two (10Me16:0 and 10Me18:0 in the R250) methyl-branched PLFAs.

The sequence analysis of DNA fragments that were excised from the DGGE gel showed the presence of the phyla *Bacteroidetes*, *Acidobacteria*, *Chloroflexi*, *Gemmatimonadetes*, *Actinobacteria* and *Betaproteobacteria*, *w*hich were also found in other heavy metal-contaminated soils (Ellis et al. 2003; Navarro-Noya et al. 2010). However, Gołębiewski et al. (2014) reported, based on the pyrosequencing results, that the bacterial communities at the level of phyla inhabiting soils that were contaminated with heavy metals were similar to those found in other non-contaminated soils, such as grassland and forest soils. In their opinion, the level of phylum, or even the class level, is not adequate for the evaluation of the differences in soil bacterial communities and that lower levels should be used for analysing the effect of heavy metals in soil.

## Conclusions

The results of presented studies show that Cd-resistant bacteria are common in long-term contaminated soils. It was also observed that the presence of *S. vulgaris* did not affect the number of colony-forming units, except for those of Cd-resistant bacteria. The abundance of Cd-resistant bacteria was affected by the distance from the emitter, which indicated that the number of these bacteria increased with an increasing contamination level. However, the presence of *S. vulgaris* altered the soil bacterial community structure, regardless of the sampling place, which supported the thesis that plants have a higher impact on a soil microbial community than metal contamination. Based on the obtained results, we are not able to postulate whether microbial communities in plant-free plots form a homogeneous group compared with plots where *S. vulgaris* is present. Further studies, which should include a larger number of sampling sites and the application of more sensitive methods (e.g. next-generation sequencing), are needed to evaluate this issue more precisely.
